# A Radiation-Crosslinked Gelatin Hydrogel That Promotes Tissue Incorporation of an Expanded Polytetrafluoroethylene Vascular Graft in Rats

**DOI:** 10.3390/biom11081105

**Published:** 2021-07-27

**Authors:** Sohei Matsuura, Toshio Takayama, Tomoko G. Oyama, Kotaro Oyama, Mitsumasa Taguchi, Takashi Endo, Takafumi Akai, Toshihiko Isaji, Katsuyuki Hoshina

**Affiliations:** 1 Division of Vascular Surgery, Department of Surgery, Graduate School of Medicine, The University of Tokyo, Tokyo 113-8655, Japan; somatsuura-tky@umin.ac.jp (S.M.); endot-sur@h.u-tokyo.ac.jp (T.E.); taka_akai@hotmail.com (T.A.); isajit-sur@h.u-tokyo.ac.jp (T.I.); traruba@gmail.com (K.H.); 2 Takasaki Advanced Radiation Research Institute, Quantum Beam Science Research Directorate, National Institutes for Quantum and Radiological Science and Technology (QST), Gunma, 1233, Takasaki 370-1292, Japan; ohyama.tomoko@qst.go.jp (T.G.O.); oyama.kotaro@qst.go.jp (K.O.)

**Keywords:** expanded polytetrafluoroethylene, vascular graft, perigraft tissue incorporation, gelatin hydrogel, radiation-crosslinking

## Abstract

A prosthetic vascular graft that induces perigraft tissue incorporation may effectively prevent serious sequelae such as seroma formation and infection. Radiation-crosslinked gelatin hydrogel (RXgel) mimics the chemical and physical properties of the in vivo extracellular matrix and may facilitate wound healing by promoting tissue organization. Fibroblasts cultured on RXgel actively migrated into the gel for up to 7 days. RXgels of three different degrees of hardness (Rx[10], soft; Rx[15], middle; Rx[20], hard) were prepared, and small disc-like samples of RXgels were implanted into rats. In vitro and in vivo results indicated that Rx[10] was too soft to coat vascular grafts. Thus, expanded polytetrafluoroethylene (ePTFE) vascular grafts coated with RXgel were developed using Rx[15] and Rx[20] gels, and ring-shaped slices of the graft were implanted into rats. Alpha-smooth muscle actin (αSMA) and type III collagen (Col-III) levels were detected by immunohistochemistry. Immunohistochemical staining for αSMA and Col-III demonstrated that RXgel-coated vascular grafts induced more granulation tissue than non-coated grafts on days 14 and 28 after implantation. RXgel-coated ePTFE vascular grafts may provide a solution for patients by reducing poor perigraft tissue incorporation.

## 1. Introduction

Prosthetic vascular grafts have been widely used in bypass surgery procedures to treat cardiovascular diseases. Perigraft tissue incorporation is an important biological process for stabilizing an implanted prosthetic vascular graft inside the host body. Poor perigraft tissue incorporation can induce serious complications such as graft infection [1,2,3,4,5] and perigraft seroma [6,7,8,9,10,11,12], in which prosthetic graft is floating in serous fluid exudated from the prosthetic graft wall. Both can result in repeat surgery, bacteremia, limb-loss, or even loss of life. Additionally, treatment of infected prosthetic grafts can be very costly [13]. Expanded polytetrafluoroethylene (ePTFE) and polyethylene terephthalate (PET) are two materials widely used for prosthetic vascular grafts; these materials have been modified over time to improve the quality of vascular grafts. For example, collagen- or gelatin-coated PET has been used to reduce the porosity of vascular grafts [14,15,16], and heparin-bonded ePTFE vascular grafts demonstrate good patency [17,18]. However, there is no commercially available vascular graft that specifically promotes perigraft tissue incorporation at this stage.

**Figure 1:**
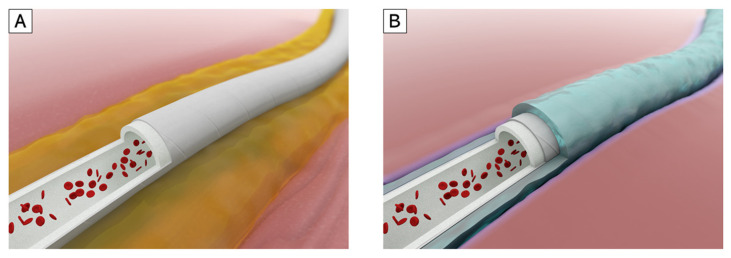
(**A**) Poor perigraft tissue incorporation can cause seroma, an abnormal fluid correction around the implanted vascular graft. Massive seroma may lead to graft infection; (**B**) A vascular graft coated with radiation-crosslinked gelatin hydrogel, a material that promotes wound healing process. We hypothesized that this coating technology may facilitate perigraft tissue incorporation.

Irradiation of polymers allows to control the characteristic and attracting attentions as a new biomaterial [19]. As previously reported by Oyama et al. [20], radiation-crosslinked gelatin hydrogel (RXgel), which can chemically and physically reproduce the native extracellular matrix (ECM), is a potential modifier for creating such a vascular graft if applied to a proper material. RXgel is capable of coating a vascular graft with containing certain amount of water to keep its ECM-mimicking property, whereas conventional gelatin-coated vascular grafts are usually provided in dry form. Gelatin is a hydrolyzed form of collagen, the main constituent of the ECM. It has relatively low antigenicity compared with collagen and is known to facilitate wound healing by recruiting tissue incorporation and organization [21,22,23]. Although the sol-gel transition of native gelatin occurs at approximately the mammalian body temperature, RXgel does not melt even at 50 °C and can reliably be used in cell culture. Unlike other chemically crosslinked gelatin hydrogels using aldehydes and carbodiimides as crosslinkers, by adjusting the dose of quantum beam irradiation using γ-rays or electron beams, an RXgel can be formed in any degree of stiffness. This covers a broad range of soft tissues without losing the natural gelatin properties such as biocompatibility, biodegradability, and cell-binding motifs [24,25]. Thus, we hypothesized that coating a vascular graft with this promising material could induce perigraft tissue incorporation promptly after graft implantation (Figure 1). We already reported the utility of RXgel as a scaffold for cell incubation [20]. The novelty of this study is that using this RXgel, we attempted to coat a medical equipment made of ePTFE, which is generally supposed to be chemically inactive and difficult to be coated.

The objectives of this study were to investigate whether RXgel itself affects the promotion of wound healing and if ePTFE vascular grafts coated with RXgel on the outer surface exhibit suitable perigraft tissue incorporation.

## 2. Materials and Methods

All animal procedures were performed according to the Guide for the Care and Use of Laboratory Animals published by the US National Institutes of Health (NIH Publication, 8th edition, 2011) [26]. All protocols were approved by the Institutional Animal Care and Use Committee of the University of Tokyo (Permit Number: M-P19-025) and were performed in accordance with the institutional guidelines as stated by the University of Tokyo. Male Sprague-Dawley rats (*n* = 116, Tokyo Laboratory Animals Science Co., Ltd., Tokyo, Japan), fed a normal diet, were used in the experiments. To ensure homogeneity in the study, we used only male rats. The animals were kept in an air-conditioned (21 ± 1 °C) and conventional environment with a 12 h light-dark cycle. In an isolation rack, there were no more than two companions, and they had ad libitum access to food and water.

During each operation, the rats were anesthetized by inhalation of 1–2% isoflurane, with concentration adjustments if slight movement was detected. All rats were euthanized by anesthesia overdose, and perfusion fixation was performed by intracardiac injection of 20 mL of 4% paraformaldehyde after pre-infusion with normal saline. 

### 2.1. Preparation of RXgel

The radiation crosslinking of gelatin, the processes of RXgel preparation, and their chemical and physical properties have been previously reported by Oyama et al. [25]. Briefly, gelatin (porcine skin, Type A, G1890; Sigma-Aldrich, St. Louis, MO, USA) solution was prepared by dissolving the gelatin in deionized water at 10 wt% with heating at 50 °C for 30 min. For cell culture, the solution was poured into cell culture dishes (φ35 mm, AGC Techno Glass, Shizuoka, Japan). The gelatin underwent physical gelation during overnight storage at 20 °C. The samples in sealed bags were irradiated with ^60^Co γ-rays (^60^Co No. 2; Irradiation Facility of Takasaki Advanced Radiation Research Institute, QST) in air at ~20 °C. RXgels were obtained after the samples were immersed in phosphate-buffered saline (PBS; pH 7.4) at 50 °C for 2 h to remove any non-crosslinked components of gelatin. The hydrogels were used for cell culture after immersion in culture medium at 37 °C for 1 h to replace the absorbed PBS with medium.

Three types of RXgels, Rx[10], Rx[15], and Rx[20], were fabricated by irradiation of 10, 15, and 20 J/g (=kGy), respectively. Higher irradiation doses generate higher crosslinking densities, resulting in a harder gel [24,25]. The compressive modulus evaluated with an indentation tester (RE2-3305B; Yamaden, Tokyo, Japan), and the water contents of the hydrogels are summarized in Table 1.

### 2.2. Fibroblast Culture on RXgel

To evaluate the ability to recruit tissue-organizing cells inside the RXgel, an invasive assay of fibroblasts on each RXgel (Rx[10], Rx[15], and Rx[20]) was performed. 3T3-Swiss albino mouse embryonic fibroblasts (RCB1642) obtained from the RIKEN BRC Cell Bank (Ibaraki, Japan) were cultured in Dulbecco’s modified Eagle’s medium (08488-55; Nacalai Tesque, Kyoto, Japan) supplemented with 10% fetal bovine serum (12483020; Thermo Fisher Scientific, Waltham, MA, USA), 100 U/mL penicillin, 100 μg/mL streptomycin (15140122; Thermo Fisher Scientific), and 2 mM L-glutamine (G7513; Sigma-Aldrich). A 2-mL aliquot of solution containing 1 × 10^4^ cells/mL was introduced onto the RXgels (formed in φ35 mm dish, ~2 mm in thickness) without any additional reagent. The cells were cultured at 37 °C in 5% CO_2_. 

On days 3 and 7 of cell culture, the samples were washed with PBS, and the cells were fixed with 4% paraformaldehyde (163-20145; Fujifilm Wako Pure Chemical Corporation, Osaka, Japan) for 15 min. After washing with PBS, the cells were incubated in PBS containing 0.1% Triton X-100 (35501-02; Nacalai Tesque) for 5 min and then washed with PBS. The cells were incubated in PBS containing 0.1 μg/mL tetramethylrhodamine B isothiocyanate-conjugated phalloidin (P1951; Sigma-Aldrich), 1 μg/mL 4′,6-diamidino-2-phenylindole (D523; Dojindo Laboratories, Kumamoto, Japan), and 1% bovine serum albumin (P6154; Biowest, Nuaillé, France) for 20 min. To visualize the surface of the hydrogels, the samples were washed with PBS and incubated for 10 min in PBS containing 0.2 mg/mL of 0.2 μm fluorescent microspheres (F8811; Thermo Fisher Scientific). The samples were then washed with PBS and examined with an upright microscope (BX51WI) with a disk scanning unit, objective lens (LUMPLFLN 60XW) (all from Olympus Corporation, Tokyo, Japan), and a complementary metal oxide semiconductor camera (ORCA-Flash4.0 V3; Hamamatsu Photonics, Shizuoka, Japan). Staining and observation were performed at ~25 °C.

### 2.3. Evaluation of RXgel Properties In Vivo

As RXgel is a biodegradable material [24,25], it is expected to be gradually absorbed and replaced by fibrous granulation tissue over time in vivo. To evaluate the ingrowth of tissue into the RXgel, we implanted Rx[10], Rx[15], and Rx[20] samples into the subfascial space of Sprague-Dawley rats (12–17 weeks old; 400–600 g, *n* = 3/group). The samples were prepared as RXgel discs (1-mm thickness and 6-mm diameter) by punching out homogenous RXgel sheets. These discs were placed in a space created between the fascia and muscle of the rectus abdominis as described by Takayama et al. [27]. On days 7, 10, and 14 after implantation, the rats were euthanized and fixed by intracardiac injection of 20 mL of 4% paraformaldehyde at 120 mmHg. The RXgel samples were explanted en bloc including the surrounding tissue and divided into two pieces in the container for embedding in paraffin. 

Next, 5-μm-thick sections were cut from each paraffin block and stained with hematoxylin and eosin, which revealed the remaining RXgel as a non-structural, rectangular region. Photomicrographs of hematoxylin and eosin-stained sections were captured and area (mm^2^) of the remaining RXgels was measured with ImageJ ver 1.4.3 software (National Institutes of Health, Bethesda, MD, USA). As the maximal cross-sectional area of the original RXgel disc was 6 mm^2^, the percentage of the remaining area of the RXgel over 6 mm^2^ was defined as the “Gel remaining ratio.”

We then performed immunohistochemical staining for alpha-smooth muscle actin (αSMA) to visualize newly formed blood vessels and recruited myofibroblasts (which are crucial elements of tissue organization) inside the implanted RXgel. After heat-induced antigen retrieval (120 °C; 5 min) and pretreatment with 100% methanol and 0.3% H_2_O_2_, a Histofine SAB-PO (M) kit (Nichirei Biosciences, Inc., Tokyo, Japan) was used according to the manufacturer’s instructions. The sections were incubated with mouse monoclonal antibodies against αSMA (1:200, M0851, Dako North America, Inc., Carpinteria, CA, USA) overnight at 4 °C, followed by staining with DAB Tablet (FUJIFILM Wako Pure Chemical Corporation). Photomicrographs of the stained sections were acquired, and a 500 × 1000-μm rectangular area at the center of the remaining RXgel was identified as the region of interest (ROI), where the αSMA-positive area (μm^2^) was measured with ImageJ software. The percentage of the αSMA-positive area compared to the ROI was defined as the “αSMA-positive ratio.”

### 2.4. Creation of RXgel-Coated ePTFE Vascular Grafts

**Figure 2:**
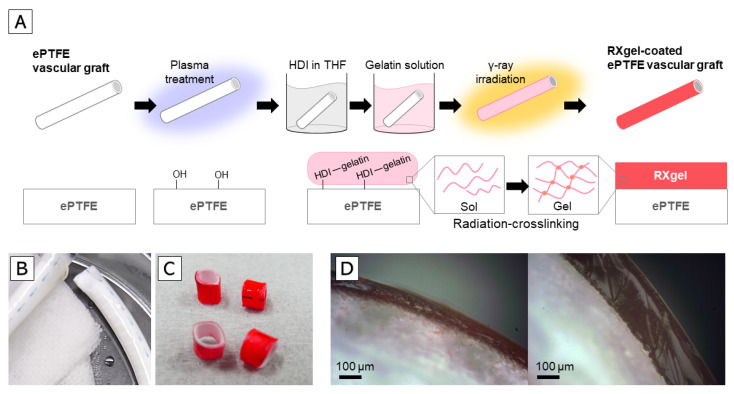
Radiation-crosslinked hydrogel (RXgel)-coated expanded polytetrafluoroethylene (ePTFE) vascular grafts based on radiation-crosslinking technique: (**A**) Schematic of fabrication process for RXgel-coated ePTFE vascular grafts; (**B**) Photograph of a typical RXgel-coated ePTFE vascular graft; (**C**) RXgel layer visualized using red food dye; (**D**) Cross-sectional images of representative RXgel-coated ePTFE vascular grafts of two different coating thicknesses.

We used a commercially available ePTFE vascular graft (AdvantaVTX, Atrium Maquet Getinge Group, Hudson, NH, USA, 4-mm diameter) to create an RXgel-coated vascular graft. The RXgel layer was chemically bonded to the outer surface of the vascular grafts (Figure 2A). First, the surface of the vascular graft was activated by irradiation with air plasma (YHS-R, SAKIGAKE-Semiconductor Co., Ltd., Kyoto, Japan) for 5 min to produce hydroxy groups on the ePTFE surface. Next, the grafts were immersed in a 1:1 solution of hexamethylene diisocyanate (HDI) and tetrahydrofuran for 1 h. After removing unreacted HDI by washing in tetrahydrofuran, the grafts were immersed in 10 wt% gelatin solution prepared as described above. The physical gelatin gel was formed around the grafts after removing the grafts from the gelatin solution. At this point, a part of the amino acids in the physical gelatin gel was chemically bonded to the vascular graft surface via HDI. The thickness of the physical gel was controlled by the number of immersions in the solution. After overnight storage at 20 °C, the grafts were irradiated with ^60^Co γ-rays. RXgel-coated vascular grafts were obtained after immersing in PBS at 50 °C for 2 h to remove non-crosslinked components of gelatin (Figure 2B). The thickness of the transparent RXgel layer chemically bonded to the ePTFE vascular graft was evaluated after immersion in red food dye for visualization (Figure 2C).

Based on our previous experience, the physical stiffness of Rx[10] is too soft for surgical manipulation. Therefore, herein, we coated the ePTFE graft with Rx[15] and Rx[20] in two thickness layers (thin or thick) to prepare four types of RXgel-coated grafts as follows:

ePTFE vascular graft coated with Rx[15] in thin layer (Rx[15]_t_)
ePTFE vascular graft coated with Rx[15] in thick layer (Rx[15]_T_)
ePTFE vascular graft coated with Rx[20] in thin layer (Rx[20]_t_)
ePTFE vascular graft coated with Rx[20] in thick layer (Rx[20]_T_)

The thickness of the layer coated on each vascular graft was measured using the cross-sectional images (Figure 2D and Table 2).

### 2.5. Evaluation of RXgel-Coated ePTFE Vascular Graft Properties In Vivo

We evaluated the incorporation of perigraft tissue in RXgel-coated ePTFE vascular grafts in rats. We prepared 1-mm-thick slices of each RXgel-coated vascular graft to create a “graft ring,” which was implanted into the subfascial space of Sprague-Dawley rats (13–14 weeks old; 400–600 g, *n* = 5/group) as described above (Section 2.3). Additionally, we implanted non-coated graft rings as controls. On days 14 and 28 after implantation, we euthanized the rats and explanted the graft ring samples en bloc with the surrounding tissue after intracardiac injection of 4% paraformaldehyde. The explanted samples were divided into two pieces along the center line of the graft ring for embedding in paraffin. 

Thereafter, 5-μm-thick sections were cut from each paraffin block and immunohistochemically stained with αSMA as described above. Immunohistochemical staining for Col-III was performed to visualize the newly formed and organized tissue outside the vascular graft ring. The sections were incubated with mouse monoclonal antibodies against Col-III (1:200, ab6310, Abcam, Cambridge, UK) after enzymatic antigen retrieval with 0.1% Proteinase K solution (Proteinase K ready-to-use, S3020, Dako). Other procedures were the same as those described for αSMA.

Photomicrographs of each immunohistochemically stained section were taken. A 200 × 50-μm rectangular area was defined along the outer surface of the graft ring wall as the ROI, where the αSMA-positive area (μm^2^) and Col-III positive area (μm^2^) were measured with ImageJ software. The percentage of the αSMA-positive area compared to the ROI was defined as the “αSMA-positive ratio.” The percentage of the Col-III-positive area compared to the ROI was defined as the “Coll-III-positive ratio.”

### 2.6. Statistical Analysis

All results are expressed as the mean ± standard deviation. Statistical analysis was performed with JMP Pro 14 statistical software (SAS Institute, Cary, NC, USA). For the results of the in vitro experiment, the significance of differences was determined using the Steel-Dwass test. For the results of in vivo experiments, the significance of differences between groups was determined by one-way analysis of variance followed by Dunnett’s test. Differences were considered significant at *p* < 0.05.

## 3. Results

### 3.1. In Vitro Vertical Migration Assay for Fibroblasts into RXgel

**Figure 3:**
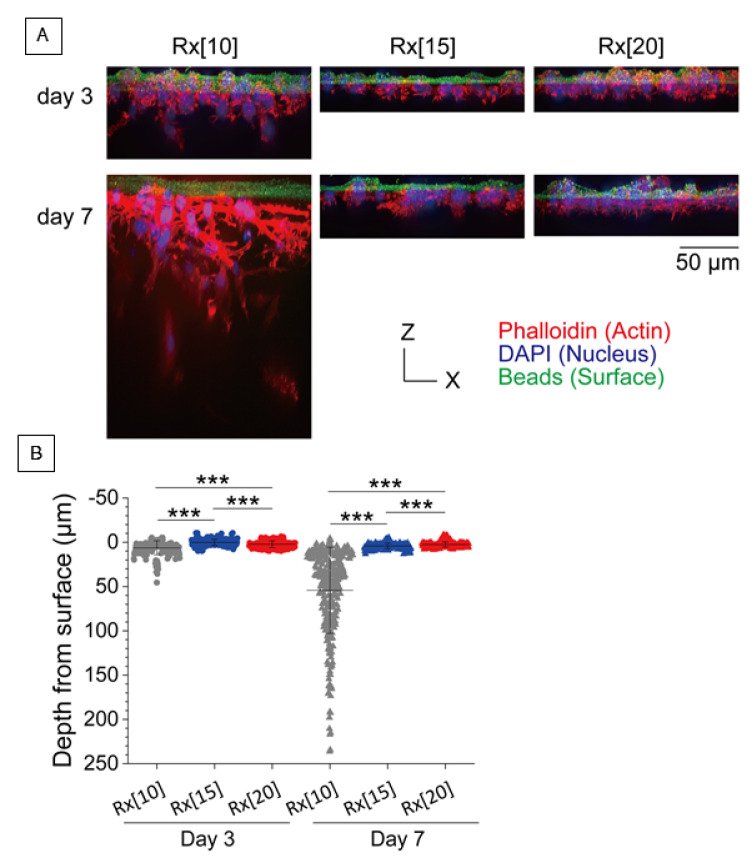
In vitro vertical migration assay for 3T3-Swiss cells into radiation-crosslinked hydrogels (RXgels): (**A**) Maximum intensity projection of confocal images of 3T3-Swiss cells on Rx[10], Rx[15], and Rx[20] grafts on day 3 (top) and day 7 (bottom). The cells were fixed and stained with TRITC-phalloidin for actin filaments (red) and DAPI for nuclei (blue). The surfaces of gels were stained with fluorescent microspheres (green); (**B**) Distribution of migrated cells in RXgels. The cells cultured on Rx[10], Rx[15], and Rx[20] for 3 days located at the depth of 6.23 ± 7.84 µm (*n* = 252 cells), 0.09 ± 3.43 µm (*n* = 251 cells), and 2.19 ± 3.84 µm (*n* = 216 cells), respectively. All values were significantly different (*p* < 0.05). The cells cultured on Rx[10], Rx[15], and Rx[20] for 7 days were located at a depth of 54.23 ± 48.65 µm (*n* = 294 cells), 4.13 ± 3.11 µm (*n* = 263 cells), and 2.48 ± 2.95 µm (*n* = 344 cells), respectively. All values were different (*** *p* < 0.0005).

To assess the stiffness of each RXgel in vitro, we observed the vertical migration of 3T3-Swiss cells cultured on Rx[10], Rx[15], and Rx[20] on days 3 and 7 (invasive assay). Although vertical migration was observed in all RXgels (Figure 3A), the depth from the surface of Rx[10] was significantly deeper among all groups (*p* < 0.0005) (Figure 3B). 

### 3.2. In Vivo Evaluation of Gel Remaining Ratio and Granulation Tissue Ingrowth into RXgel

**Figure 4:**
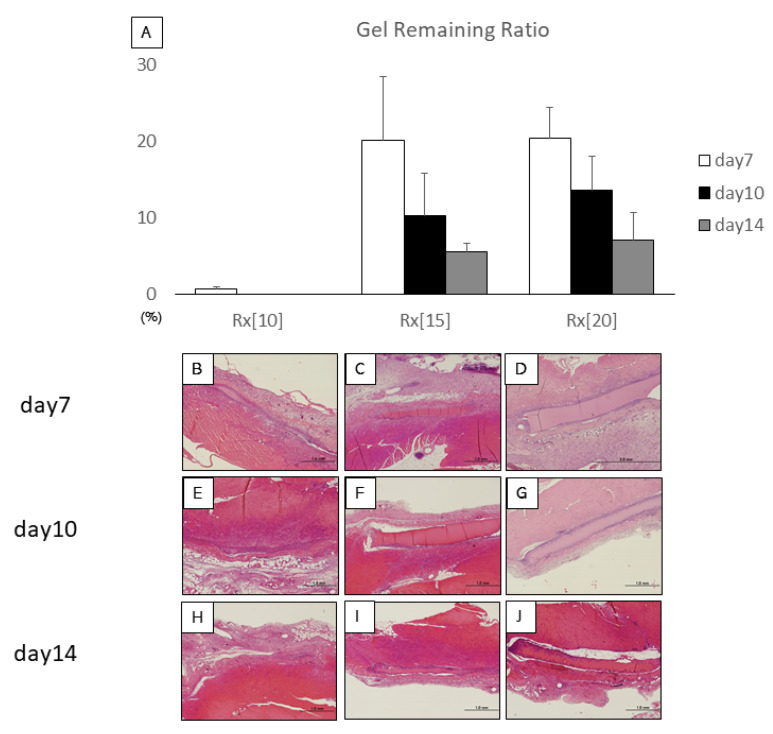
Hematoxylin and eosin (HE)-stained sections of implanted radiation-crosslinked hydrogels (RXgels): (**A**) Gel remaining ratioof RXgel analyzed by HE staining. There was no significant difference among other days in the same RXgel group; (**B**–**J**) HE staining of 5-µm-thick sections on days 7, 10, and 14 after implantation of each RX-gel. With HE staining, RXgel was seen as a non-structural substance. Each scale bar indicates 1.0 mm.

The gel remaining ratio reflects the degradation speed of RXgel in vivo. We compared the results on days 10 and 14 with that on day 7 in each gel group. All the gels appeared to degrade over time. Rx[10] almost completely disappeared on day 7, while Rx[15] and Rx[20] showed similar degradation trends. There was no significant difference between days within the same sample group though (Figure 4).

**Figure 5:**
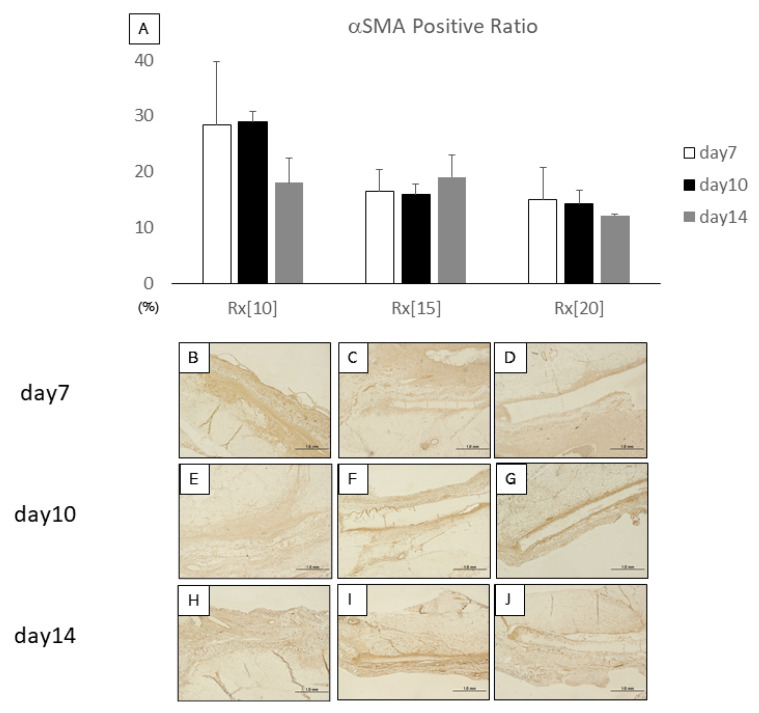
α-Smooth muscle actin (αSMA)-stained sections after implantation of radiation-crosslinked hydrogels (RXgels): (**A**) Expression ratio of αSMA was analyzed by αSMA staining. There was no significant difference between other days in the same RXgel group; (**B**–**J**) αSMA staining of 5-µm-thick sections at days 7, 10, and 14 after implantation of each RXgel. αSMA-positive areas are shown in dark brown. Each scale bar indicates 1.0 mm.

The αSMA positive ratio reflects the amount of newly formed blood vessels and recruited myofibroblasts inside the implanted RXgel samples. No significant difference between days within the same sample group was observed when results for days 10 and 14 with the result for day 7 in each gel group were compared. Whereas αSMA expression in Rx[10] tended to decrease over time, Rx[15] and Rx[20] showed rather more stable expression of αSMA up to day 14 (Figure 5).

### 3.3. In Vivo Evaluation of Tissue Ingrowth around RXgel-Coated ePTFE Vascular Grafts

Both the in vitro and in vivo results indicated that the stiffness of Rx[10] was too soft to be a potential material for vascular graft coating. We therefore created RXgel-coated ePTFE vascular grafts with Rx[15] and Rx[20].

**Figure 6:**
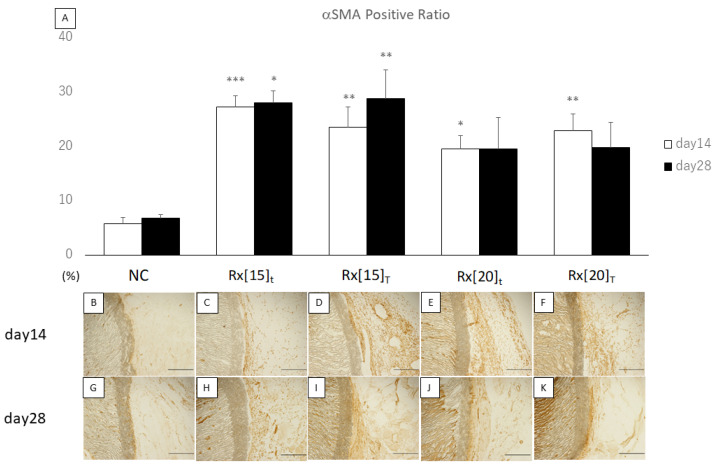
α-smooth muscle actin (αSMA)-stained sections after implantation of radiation-crosslinked hydrogel (RXgel)-coated expanded polytetrafluoroethylene (ePTFE) vascular grafts: (**A**) Expression ratio of αSMA was analyzed using αSMA staining. The significance compared to non-coated ePTFE on the same day is shown by asterisks (* *p* < 0.05, ** *p* < 0.005, *** *p* < 0.0005); (**B**–**K**) αSMA staining of 5-mm-thick sections on days 14 and 28 after implantation of each RXgel-coated ePTFE graft. In each picture, the outer surface of the ePTFE graft is in the center and at the right side of the graft wall. The αSMA-positive areas are shown in dark brown. Each scale bar indicates 100 µm.

We compared the quantitative results of the αSMA positive ratio with that of the non-coated (NC) vascular graft group on the same day. On day 14, all RXgel-coated vascular grafts showed significantly higher expression of αSMA compared to the NC grafts. On day 28, only Rx[15], regardless of the thickness, showed significantly higher expression of αSMA compared to the NC (Figure 6).

**Figure 7:**
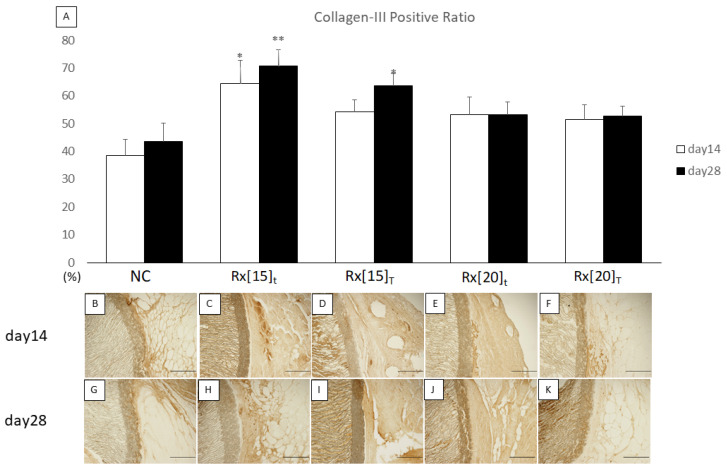
Col-III stained sections after implantation of radiation-crosslinked hydrogel RXgel-coated expanded polytetrafluoroethylene ePTFE vascular grafts: (**A**) Expression ratio of Col-III was analyzed by Col-III staining. The significance compared to non-coated ePTFE on the same day is shown by asterisks (* *p* < 0.05, ** *p* < 0.005); (**B**–**K**) Col-III staining of 5-μm sections on days 14 and 28 after implantation of each RXgel-coated ePTFE graft. In each picture, the outer surface of ePTFE graft is in the center and at the right side of the graft wall. Col-III positive areas are shown in dark brown. Each scale bar indicates 100 µm.

We also compared the quantitative results of the Col-III positive ratio with those of the NC group on the same day. On day 14, only Rx[15]_t_ showed significantly higher expression of Col-III compared to the NC, whereas on day 28, both Rx[15]_t_ and Rx[15]_T_ showed significantly higher expression of Col-III compared to the NC (Figure 7).

## 4. Discussion

The process of perigraft tissue incorporation begins as soon as a vascular graft is implanted, and the proliferative stage of wound healing continues for up to approximately 14 days after implantation [28,29,30]. Theoretically, a graft-coating material should last for at least 14 days in vivo and be absorbed and replaced by granulation tissue gradually over time. First, we compared three RXgels fabricated in different stiffnesses (i.e., crosslinking density): Rx[10] as the softest and Rx[20] as the hardest. We implanted small disc-like samples of each RXgel into rats. Rx[10] had almost disappeared as early as day 7 after implantation, whereas both Rx[15] and Rx[20] remained at similar absorbance ratios up to day 14. The gels were replaced by cell-rich granulation tissue growing inward from the surrounding host tissue. We immuno-stained the histological sections to detect αSMA expression because this protein is a major marker of myofibroblasts [31], a crucial component of the wound healing process [32]. The induced granulation tissue was αSMA-positive, and both Rx[15] and Rx[20] showed stable αSMA expression up to day 14. We also evaluated RXgels recruitment of fibroblasts by an invasive in vitro assay, which demonstrated that fibroblasts rapidly migrated deep inside Rx[10] on day 7, whereas both Rx[15] and Rx[20] dependably contained fibroblasts. These results indicate that the RXgel itself possesses a promising wound-healing ability and Rx[15] and Rx[20] are good candidates as vascular graft coating materials.

Next, we created RXgel-coated vascular grafts using Rx[15] and Rx[20] and investigated their tissue incorporation ability. Of two major vascular graft materials, we chose ePTFE over PET because ePTFE vascular grafts are widely used in arteriovenous graft blood access procedures for patients with end-stage chronic kidney disease depending on regular hemodialysis. As seroma formation after arteriovenous graft procedure is a serious problem in such patients [33], establishing a new technology to promote efficient, rapid perigraft tissue incorporation of an ePTFE graft will substantially improve their quality of life. We created four types of RXgel-coated vascular graft using two RXgels with two thickness coating layers. We implanted ring-shaped samples of each graft, including non-coated ones as controls, into rats, and observed long-term effects up to day 28 post-implantation. To determine the ability to incorporate perigraft tissue, we immuno-stained the histological sections to detect αSMA and Col-III expression, considering that αSMA expression reflects the recruitment of myofibroblasts in the early stage of wound healing [32], and Col-III is the predominant subtype of collagen at the beginning of fibroplasia [28]. Rx[15]-coated grafts showed significantly higher expression of both αSMA and Col-III compared with non-coated grafts, whereas Rx[20]-coated grafts showed a similar tendency, but the difference was not significance.

Perigraft tissue incorporation proceeds in conjunction with the wound healing process, which is divided into three stages: inflammatory, proliferative, and remodeling [28,34]. The inflammatory stage begins as soon as the host tissue is wounded [34]. The damaged capillaries trigger fibrin and fibronectin network formation, which is a provisional matrix that enables the migration of inflammatory cells, including neutrophils and macrophages, from the surrounding microenvironment [28,29]. These inflammatory cells then release cytokines that convert fibroblasts into myofibroblasts, which play an important role in the proliferative stage [31]. Myofibroblasts specifically provide stress fibers containing αSMA to form granulation tissue inside the lesion, and subsequently disappear toward the end of this stage by apoptosis [35]. In the remodeling stage, collagen secreted from the extracellular matrix is deposited in the granulation tissue to form a sturdy fibrotic tissue to complete the wound healing process [28,34]. Perigraft tissue incorporation is a favorable form of wound healing after a prosthetic vascular graft is implanted.

An ideal material for inducing perigraft tissue incorporation should not only be able to facilitate the wound healing process but also confer other important conditions such as safety (biologically safe), availability (inexpensive, easy to obtain), applicability (can be applied in a variety of forms), and biodegradability (should disappear at an appropriate time). Gelatin, the major component of RXgel, is biocompatible and biodegradable in nature, and reasonably satisfies most of these conditions including safety and availability issues [21], given that gelatin-coated vascular grafts have been widely used in cardiovascular surgery for a long time [14,36]. The unique advantages of RXgel are associated with its ECM-mimicking properties and versatile applicability. RXgel comprises only ECM-derived gelatin and water and can be fabricated at any stiffness covering a broad range of soft tissues without losing the biological functionalities of gelatin, such as biocompatibility, biodegradability, and cell-binding motifs [20,25]. This material is obtained by a simple yet effective radiation-crosslinking technique using γ-ray or electron beam irradiation, which is commonly used to sterilize food and medical products, and its stiffness can arbitrarily be altered by adjusting the dose of irradiation. The required irradiation dose for obtaining RXgel-coated vascular grafts is within the radiation sterilization dose range (4–25 J/g) [37]; that is, the irradiation process not only induces cross-linking but also sterilizes the sample. In our study, unlike non-coated ePTFE vascular grafts, RXgel-coated vascular grafts were covered with αSMA- and Col-III-rich granulation tissue by day 28 after implantation. This finding implies that RXgel is a promising material for facilitating perigraft tissue incorporation of ePTFE vascular grafts.

There are certain limitations in this study. First, our animal model could not assess the compliance mismatch between the vascular graft and host native vessels, despite the fact that we were aware of such mismatch is a well-known factor of graft failure [38,39,40,41]. Second, our animal model could not also assess whether the outer surface RXgel-coating might affect the luminal area in the long term, although we speculate that the perigraft tissue incorporation force would not strong enough to penetrate the graft wall. Third, as the nature of our rat-model based results, we have to be careful to apply the positive wound-healing effect of our vascular graft to the clinical setting. It is known that perigraft wound healing in animal models are often better than those in human subjects. Further investigation such as a bypass grafting experiment using larger animal models would be desirable to address these points.

## 5. Conclusions

RXgel, a radiation-crosslinked gelatin hydrogel, induces granulation tissue rich in myofibroblasts, and coating outer surface of an ePTFE vascular graft with RXgel facilitates organized tissue formation around the graft in a rat model. RXgel-coated ePTFE vascular grafts promote perigraft tissue incorporation after implantation, which can ultimately benefit patients by reducing serious comorbidities, such as perigraft seroma and vascular graft infection.

## 6. Patents

T.T., K.H., S.M., T.G.O., K.O., and M.T. are co-inventors on a patent related to this study.

## Figures and Tables

**Table 1:** Typical properties of prepared radiation-crosslinked hydrogels (RXgels).

	Rx[10]	Rx[15]	Rx[20]
**Compressive modulus (kPa)**	23.4 ± 2.9	66.8 ± 4.8	108.3 ± 6.1
**Water content (%)**	90.4 ± 0.6	88.7 ± 0.7	88.5 ± 0.1

Rx[10], Rx[15], and Rx[20] indicate irradiation of 10, 15, and 20 J/g (=kGy), respectively. Data represent mean ± standard error of the mean for *n* = 3.

**Table 2:** Thickness of radiation-crosslinked hydrogel (RXgel) layer coated on vascular grafts.

Sample	Rx[15]_t_	Rx[15]_T_	Rx[20]_t_	Rx[20]_T_
**Thickness [m]**	66.5 ± 1.5	179.8 ± 9.5	67.6 ± 1.3	193.3 ± 5.9

T and t indicate the Thick and thin coatings of the expanded polytetrafluoroethylene graft, respectively. Rx[10], Rx[15], and Rx[20] indicate irradiation of 10, 15, and 20 J/g (=kGy). Data represent mean ± standard error of the mean for *n* = 3.

## Data Availability

Data sharing not applicable.
